# Man With a Leaking Umbilical Hernia

**DOI:** 10.1016/j.acepjo.2026.100540

**Published:** 2026-11-02

**Authors:** Janie Hubbard, James C. Crosby, Madeline Palmer, Erin F. Shufflebarger

**Affiliations:** Department of Emergency Medicine, University of Alabama at Birmingham Heersink School of Medicine, Birmingham, Alabama, USA

**Keywords:** POCUS, surgical emergency, strangulated hernia, emergency ultrasound, small bowel obstruction

## 1 Patient Presentation

A 70-year-old man with a history of decompensated cirrhosis requiring weekly paracentesis and an umbilical hernia presented to the emergency department with 1 week of progressive umbilical hernia enlargement with umbilical drainage and overlying skin discoloration, nausea, vomiting, and decreased bowel movements. On arrival, he was afebrile and hemodynamically stable. Abdominal examination revealed distension and a protruding, tense, nontender, erythematous umbilical hernia with overlying skin necrosis and leakage of ascitic fluid (Fig). Point-of-care ultrasound (POCUS) confirmed the diagnosis (Video 1) and expedited definitive management.

**Figure:**
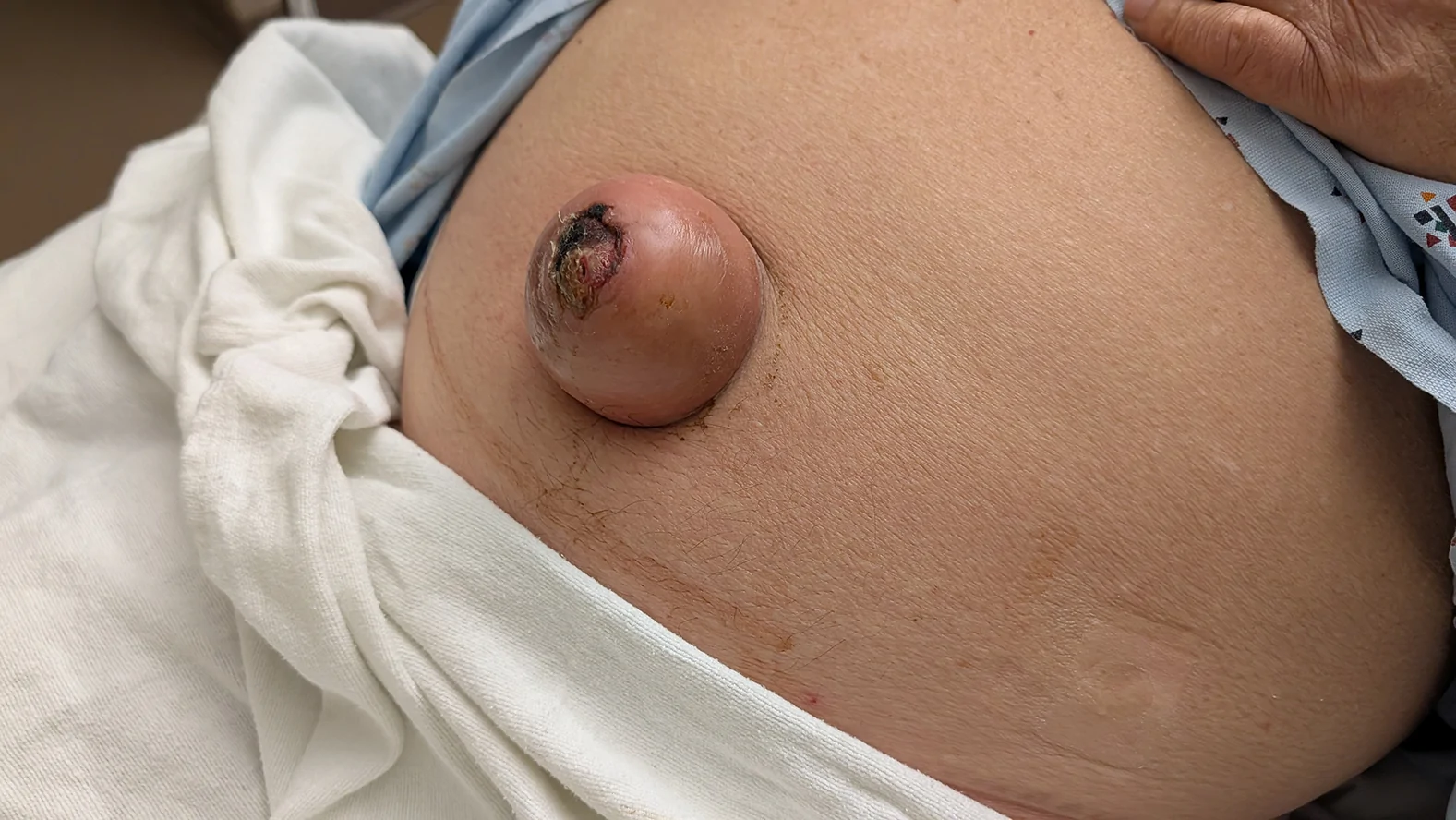
Distended abdomen with a protruding umbilical hernia, associated discoloration, and overlying skin necrosis.

**Video:**
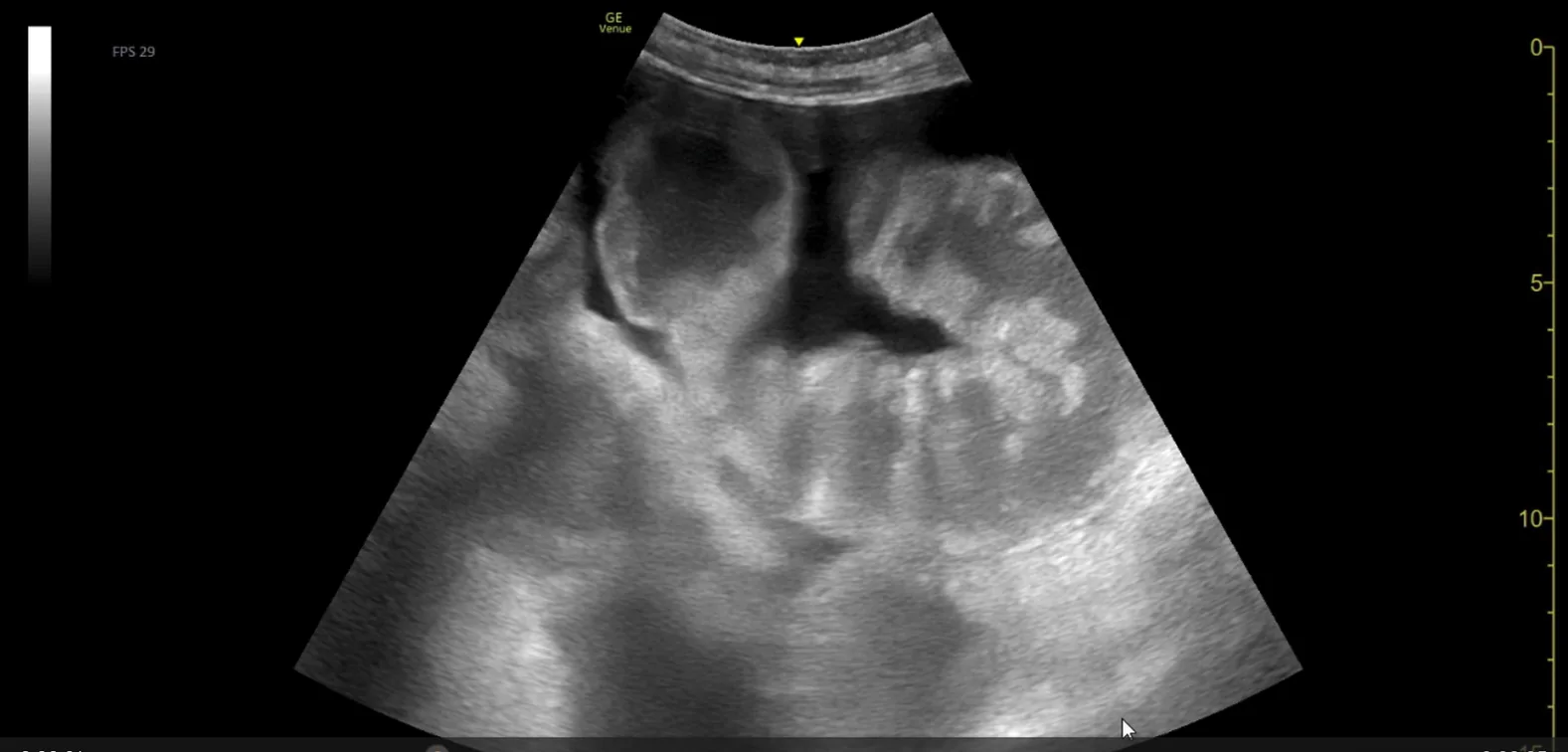
Point-of-care ultrasound clip showing ascites, dilated loops of bowel, and “to-and-fro” movement of bowel contents.

## 2 Diagnosis: Small Bowel Obstruction Secondary to Strangulated Hernia

POCUS demonstrated dilated bowel loops and “to-and-fro” movement of bowel contents (Video), consistent with small bowel obstruction (SBO) secondary to a strangulated umbilical hernia. General surgery was consulted immediately, and the patient underwent emergent operative intervention with resection of ischemic bowel.

Hernias account for approximately 10% of SBO cases.^1^ In patients with SBO, hernia-related obstruction carries a substantially higher risk of strangulation than adhesive disease (33.3% vs 9.0%).^2^ When bowel necrosis occurs, mortality increases from 10% to 30%.^1^ Delays in operative management of emergent hernias are associated with higher rates of wound infection, postoperative sepsis, prolonged operative times, and longer hospitalizations.^3^ POCUS offers a rapid bedside alternative to computed tomography and has demonstrated reasonable accuracy for the diagnosis of SBO.^4^ Sensitive sonographic findings include small bowel dilation ≥25 mm and abnormal peristalsis.^5^ In this case, the combination of concerning examination findings and POCUS evidence of bowel obstruction enabled prompt diagnosis and expedited surgical intervention.

## Ethics declaration

Written informed consent for all photographic images and to publish the article has been obtained from the patient. The privacy rights of participants have been observed.

This study was performed in compliance with relevant laws, regulatory frameworks and guidelines where the research took place. Ethics committee approval was not required under relevant laws and institutional guidelines. Per POL043: "UAB has determined, based on review of the regulations, that a case report of three (3) or fewer cases (with an n < 3) does not constitute human subjects research and does not require review by the UAB IRB."
